# Supplementary material for: Reconstructing missing complex networks against adversarial interventions
Source: Nat Commun. 2019 Apr 15;10:1738. doi: 10.1038/s41467-019-09774-x (PMC6465316; doi:10.1038/s41467-019-09774-x)
Supplement: Supplementary file 1 — Supplementary Information [file 41467_2019_9774_MOESM1_ESM.pdf]

Supplementary Materials for Reconstructing Missing Complex Networks against Adversarial Interventions

Yuankun Xue[1] **and Paul Bogdan**[1,*]

[1]Ming Hsieh Department of Electrical and Computer Engineering, University of Southern California, CA, USA, 90007
[*]Correspondence author, Email: pbogdan@usc.edu

Supplementary Materials for Reconstructing Missing Complex Networks against Adversarial Interventions

Yuankun Xue[1] **and Paul Bogdan**[1,*]

[1]Ming Hsieh Department of Electrical and Computer Engineering, University of Southern California, CA, USA, 90007
[*]Correspondence author, Email: pbogdan@usc.edu

Supplementary Note 1: A motivating example to show causal transition structure under network interventions

Consider the following intervention model,

$$\mathscr{A}_\alpha(d_i,t) = \frac{d_i^\alpha}{\sum_i^{N(t)} d_i^\alpha} \tag{1}$$

A critical observation on Equation (1) refers to the *causal* dynamics of interventions. The distribution $\mathscr{A}_\alpha(d_i,t)$ of interventions leads to time-varying behavior as a function of G_t. From a dynamic perspective, this time-varying distribution of the intervention leads to a time-inhomogeneous Markovian transition of G_t between different configurations in time: At a time point s, the attacker makes a stochastically greedy choice that prioritizes its preference on the nodes degree (e.g., either hub-preferential when $\alpha > 0$ or the opposite when $\alpha < 0$), which has *no* explicit memory on the trajectory of the interventions in the past given the network G_s at that moment. However, G_s is a causal consequence of all prior intervention sequences. Effectively, this means a dynamic Markovian transition structure from G_s to G_{s+1} where $P_{s,i,j}$ is a $\binom{N}{s}$ by $\binom{N}{s+1}$ transition probability matrix (assuming only one node removed at each time step). $P_{s,i,j}$ follows the Markov property yet has time-varying state space and time-inhomogeneous transition probability (given by $A_\alpha(d,s)$). To better understand this, consider the example of a 4-node network under the 2-step intervention in Supplementary Figure 1.(b) and assume hub-prioritized intervention ($\alpha > 0$). G_0 is the original network and G_2 is what we observe. Nodes A and B are missing because of the intervention. In this simple example, two trajectories from G_0 to G_2 exist, namely, $A \to B$ or $B \to A$. However, by Equation (1), only $A \to B$ is possible as the attacker chooses only connected nodes. Clearly, G_2 is a consequence of the attack performed in sequence and there is a causal link in time between consecutive transitions. Inferring G_0 from G_2 requires the identification of such causal interdependency introduced by the time-varying (relative) preference of the attacker, although the same intervention strategy is maintained all the time (i.e., α is not a function of time).

Supplementary Note 2: Analysis of small world property of example real networks

Network	C_Δ	C_{ws}	L	$\bar{C}_r$	$\bar{L}_r$	S_Δ	S_{ws}	$\lvert G\rvert$	$\lvert E\rvert$
facebook	0.5013	0.5406	3.0660	0.0109	2.1828	35.2577	32.6937	4049	91575
hu.Map	0.6432	0.3651	9.6820	0.0034	3.1362	61.2782	34.7834	4035	28366
brain	0.1069	0.3047	3.4879	0.0105	3.5924	10.5103	29.9697	1015	1000

Supplementary Table 1. Quantification of small-worldness of the real networks.

Small world networks are usually identified and defined from two major structural signatures, namely, much higher clustering coefficient and similar average shortest path length as compared the Erdos-Renyi random graphs. The clustering coefficient of a network measures the extent to which the neighbors of a node are also interconnected. In practice, there are two (not equivalent) ways to compute the clustering coefficient of a network. The first definition of the clustering coefficient of a graph is an average of the local clustering coefficient computed for each node in the network[1]. Given a node i, its clustering

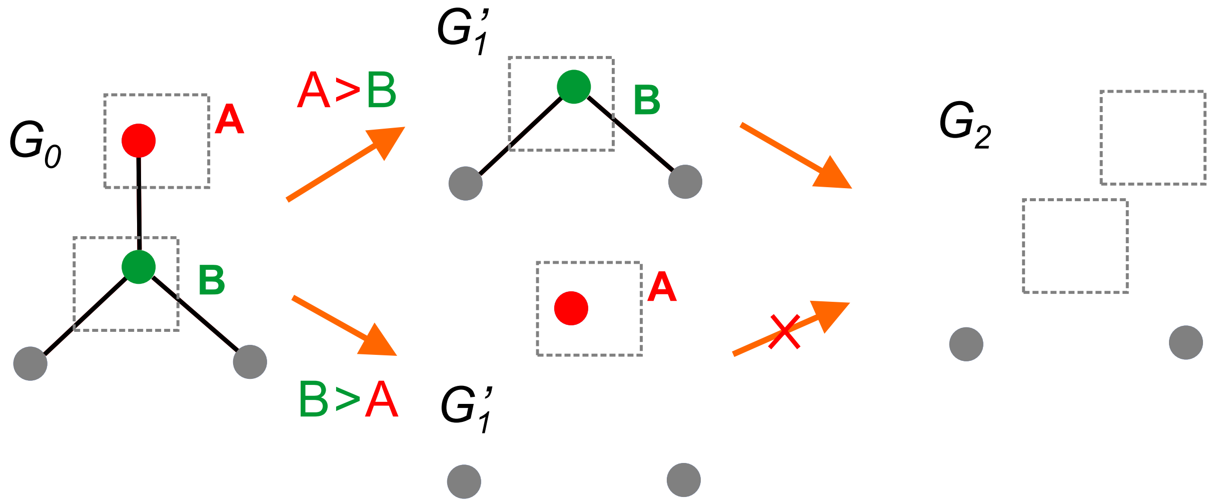

Supplementary Figure 1. A motivating example. Temporal causal transition structure of G_t under time-varying interventions. G_s and $G_{s'}$ $(s < s')$ are causally interdependent even though the adversarial party always makes Markovian decisions.

coefficient is computed by,

$$c_{ws}(i) = \frac{2E_i}{k_i(k_i - 1)} \tag{2}$$

where E_i is the number of existing edges between neighbors of node i and k_i is number of its immediate neighbors. Therefore, the local clustering coefficient $c_{ws}(i)$ measures the density of edges between neighbors of node i. The global clustering coefficient C_{ws} thus can be computed by the average of $c_{ws}(i)$. An alternative definition of clustering coefficient C_Δ in common use[2], based on transitivity, is computed by,

$$C_\Delta = \frac{3N_\Delta}{N_{l=2}} \tag{3}$$

where N_Δ is the number of triangles and $N_{l=2}$ is the number of paths of length 2. A triangle is a set of three nodes in which each directly connects to the other two. Both C_{ws} and C_Δ quantify the density of interconnections of neighbors of a node in the average sense whereas they are not mathematical equivalent. Therefore, we report the small-worldness of our considered networks based on these two different definitions. A common quantitative definition of a small-world network is stated as follows[3],

Definition 1 (Small-worldness): A network is said to be a small-world network if $S > 1$ where S is defined as,

$$S = \frac{\gamma}{\lambda} \tag{4}$$

$$\gamma = \frac{C}{C_r} \tag{5}$$

$$\lambda = \frac{L}{L_r} \tag{6}$$

where C and C_r is the clustering coefficient of the considered graph and its equivalent Erdos-Renyi random network. An equivalent Erdos-Renyi random network of a given network of size N is obtained by randomly assigning its edges to pairs of nodes in an empty network of same size. C by our definition can be C_{ws} or C_Δ. For Erdos-Renyi random network, the expectation of C_r can be well approximated by $< d > /N$ where $< d >$ is its average degree[4]. L is the average of the length of all-pair shortest paths. The expectation of L_r is computed by $\ln(N)/\ln(< d >)$ for a Erdos-Renyi random network. Therefore, the S serves as a measurement of small-worldness of a network. Intuitively, a bigger S suggests that the network is closer to a small-world network. Following the definition, a network is a small-world network if $S > 1$. To verify whether the networks (facebook, hu.Map and human brain connectome) in our experiments are small-world networks, we compute S for each network based on two definitions of C and report the results in Supplementary Table 1. For all networks, S (S_ws or S_Δ) is much greater than 1 and ranges from 10.5103 to 64.2782. This suggests that all three networks are small-world network,

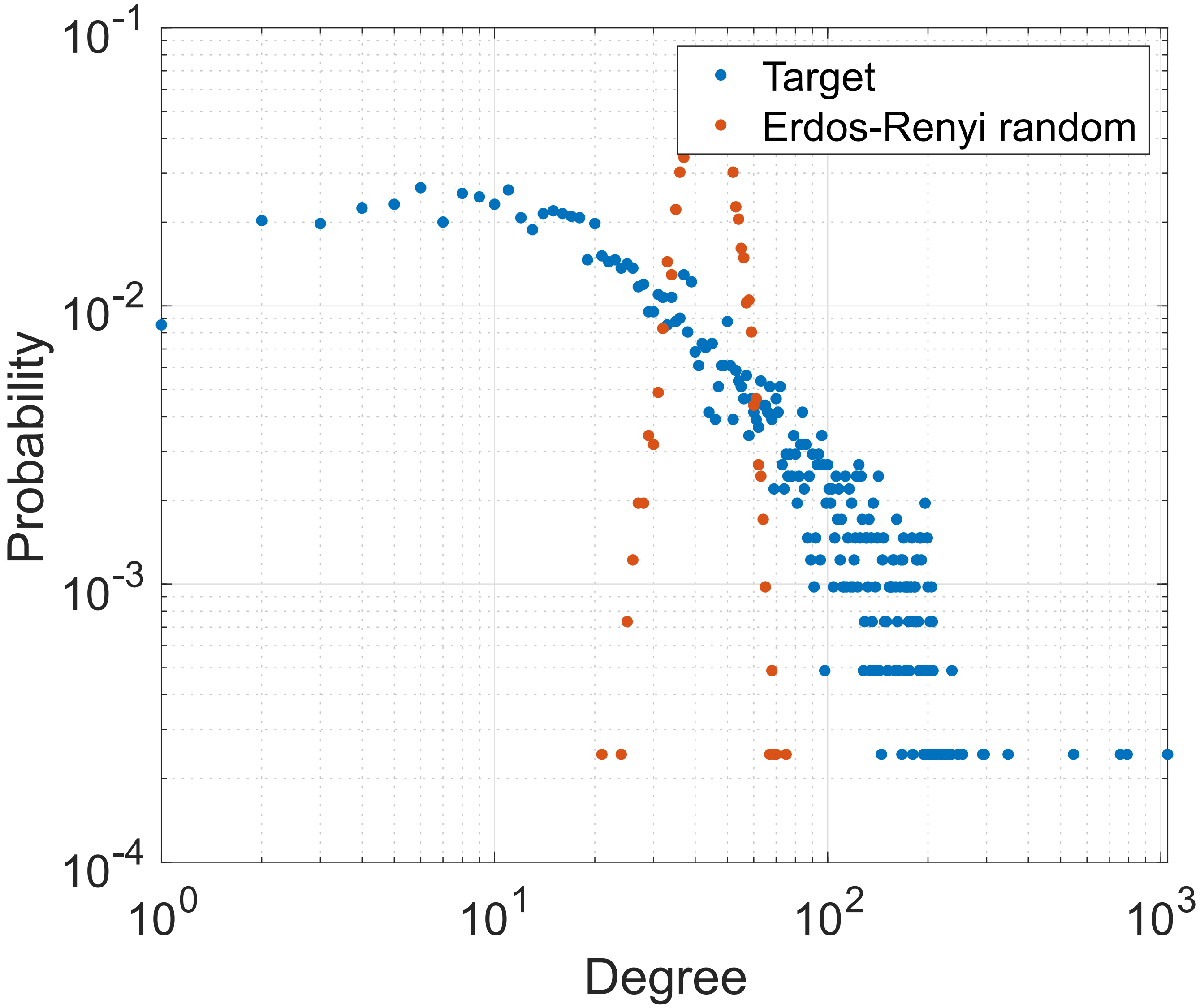

Supplementary Figure 2. Degree distribution of the facebook social network and its equivalent Erdos-Renyi random network.

hence following its structural robustness against random removals. In addition, small-world network usually has a greater than expected number of hubs (as compared to its Erdos-Renyi equivalents) and thus has a greater fraction of nodes with high degree. Consequently the degree distribution will be enriched at high degree values (i.e., long or fat tailed). To further corroborate our discussion, we visualize the degree distribution (Target) of three networks in Supplementary Figure 2-4. To show the difference, we also plot the degree distribution of an instance of their Erdos-Renyi equivalents (Erdo-Renyi random). As we can see from these figures, there is a considerable greater amount of probability mass concentrated on the region of high degrees, as opposed to that of their Erdos-Renyi equivalents, indicating the more-than-expected existence of hub nodes. More importantly, we notice a linear dependence in Supplementary Figure 2 and Supplementary Figure 3, suggesting that they are also scale-free networks where the degree distribution obeys a power-law. Scale-free networks are actually ultra small-world networks[5].

Supplementary Note 3: Transitional behavior of interventions

All three networks in the experiments are small-world networks with a small portion of its nodes significantly more connected than the rest. A hub-prioritized intervention therefore changes its statistical behavior over time as the hubs are being removed. It changes from a highly targeted removal to a close-random removal. To verify this, we have shown the empirical CDF (eCDF)

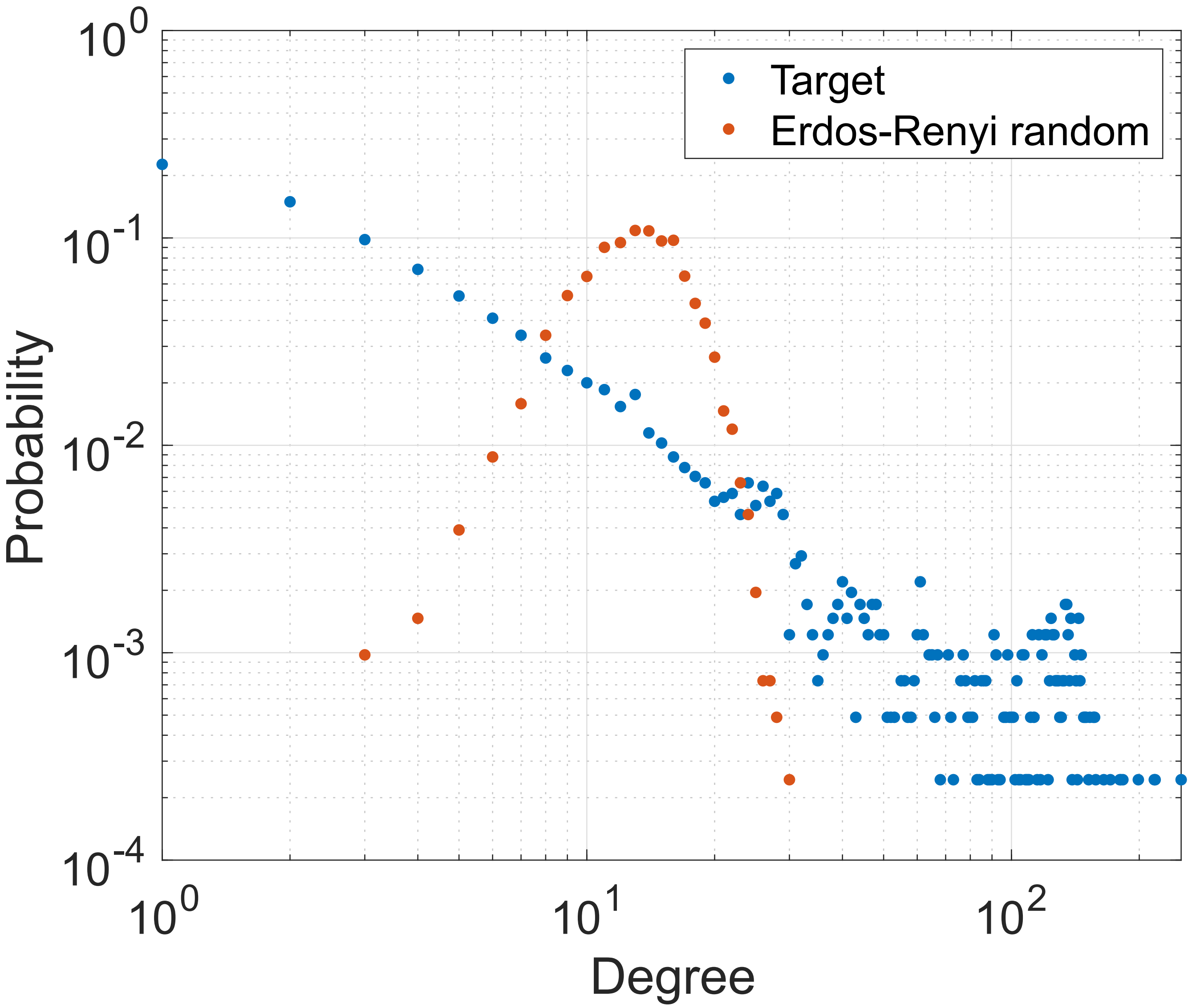

Supplementary Figure 3. Degree distribution of the human protein complex interaction network and its equivalent Erdos-Renyi random network.

of how a strongly hub-prioritized ($\alpha = 10$) attacker chooses its target over time in Supplementary Figure 5-7. If the attacker purely randomizes its choice irrespective of target's degree, we will observe the blue dashed line being a linear function of the node ID, implying a uniform distribution. Otherwise, if certain nodes are significantly preferred than others, we will observe large jumps in the eCDF curves, an indicator of strong preference. Supplementary Figure 5 exactly shows such patterns over the course of the intervention. Initially, hub nodes are not all removed (e.g., 1st step or 5-th step) and the intervention strategy is highly prioritizing the hubs. As hubs being removed, the eCDF changes close to the dashed line, suggesting that the attacker chooses almost randomly from the residual network. Similar observations are due in Supplementary Figure 6 and Supplementary Figure 7, which support our analysis made in the manuscript.

Supplementary Note 4: Multi-fractal Network Generative Model

We consider the multi-fractal network generative model (MFNG) due to its capability to reproduce the properties of a wide range of real networks and its theoretical roots in the thermodynamic limit of graph sequences. A sequence of network G_n is convergent if the sequence $t(F, G_n)$ has a limit for every simple network F. $t(F, G)$ is the homomorphism density defined as:

$$t(F,G) = \frac{hom(F,G)}{|V(G)|^{V(F)}} \tag{7}$$

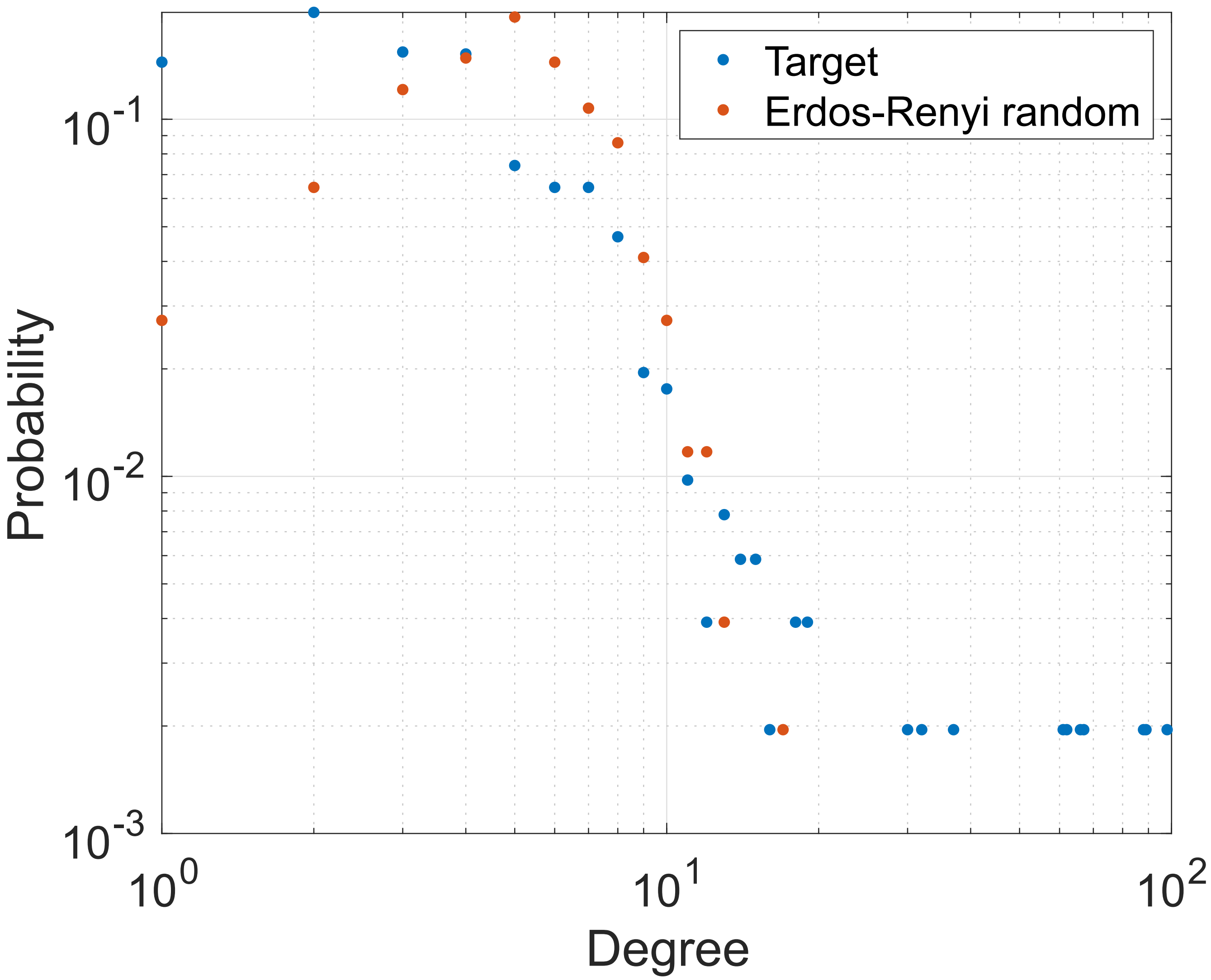

Supplementary Figure 4. Degree distribution of the human consensus brain connectome and its equivalent Erdos-Renyi random network.

$hom(F,G)$ is the total number of adjacency preserving mapping between F and G. That is, for every edge $e_{i,j}$ in $E(F)$, the edge $e_{\psi(i),\psi(j)}$ is in $E(G)$. $E()$ and $V()$ take the edge and node sets, respectively. For instance, $hom(F,G) = |V(G)|$ if F is a single node.[6] proves that any convergent graph sequence can be represented by a symmetric measurable 2D function $0 \leq W(x,y) \leq 1$ defined on the unit square. This 2D function gives the linking probability for pair of nodes randomly mapped to $[0,1]$. The average degree of a network generated from predefined $W(x,y)$ can thus be derived by

$$< d >= N \int \int W(x,y) dxdy \tag{8}$$

Intuitively, $W(x,y)$ can be understood as a probability measure that quantifies the linking probability of two nodes contained in the box defined by $[x, x+dx]$ and $[y, y+dy]$. The probability to find two nodes in this box is equal to the area of the box (as they are defined on a unit square). It is noted that in the limit that N becomes infinity, $< d >$ is also unbounded, suggesting the graph converges to a dense graph in contrast to the fact that real networks are usually sparse. To account for the sparsity of real networks, MFNG replaces the $W(x,y)$ with a multi-fractal measure defined on the unit square.

Let $\mathscr{G}_k = (m, k, \mathscr{P}_0, \mathscr{L})$ be the multi-fractal network generative model (MFNG). A network G is a realization of $\mathscr{G}_k$ if G is sampled from the following stochastic procedure:

(1) A MFNG $\mathscr{G}_k = (m, k, \mathscr{P}_0, \mathscr{L})$ partition the interval $[0,1]$ into m intervals of length $\mathscr{L} = \{l_1, l_2, ..., l_m\}$ where $\mathscr{L}$ forms

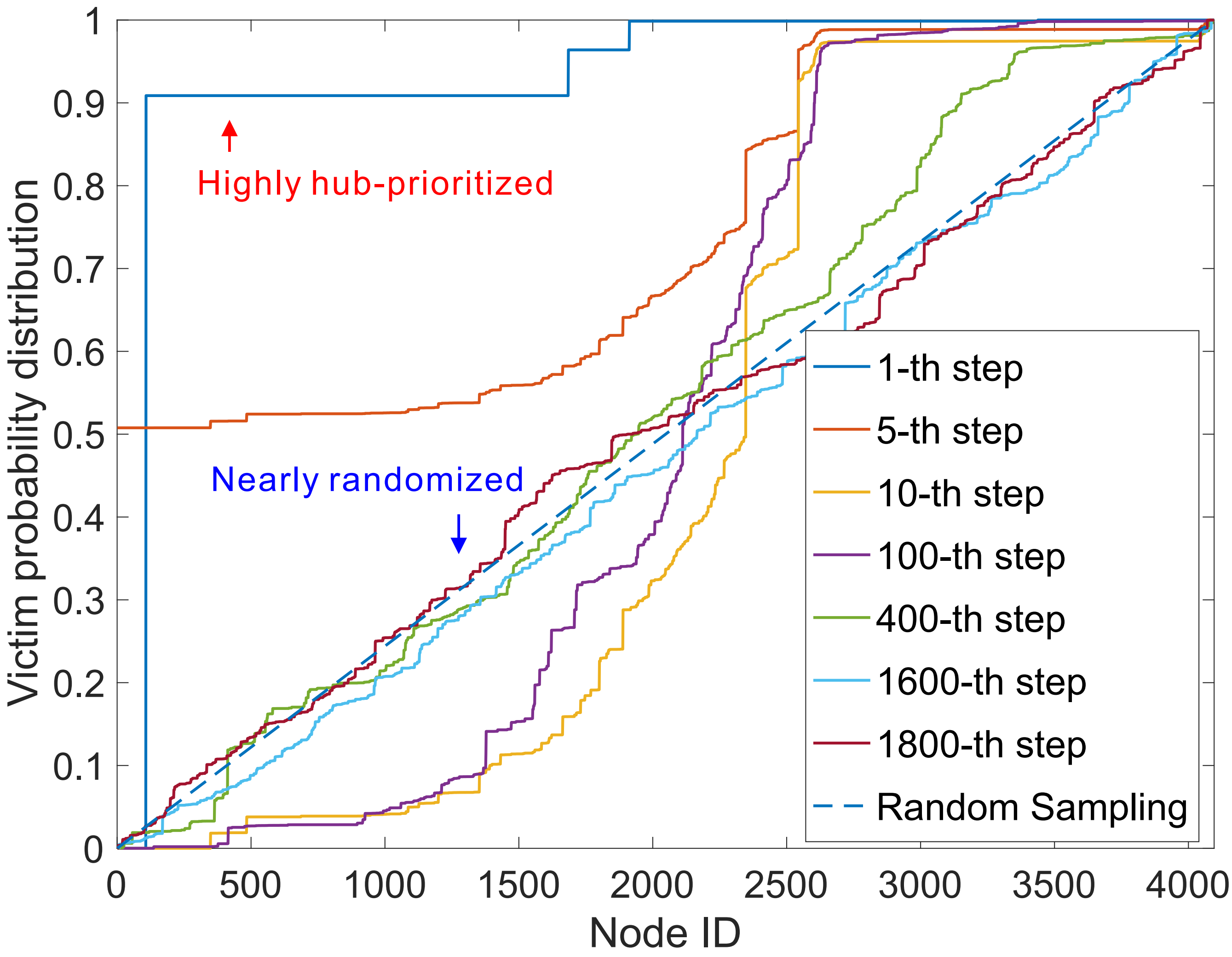

Supplementary Figure 5. Transitional behavior of interventions ($\alpha = 10$) on the facebook social network over time.

a categorical distribution over interval $[0,1]$ such that:

$$P\{S^{(1)}(x) = i\} = l_i, x \in [0,1] \tag{9}$$

where $S^{(i)} : \mathscr{R} \rightarrow \{1,2,...,m\}^i$ is an indexing function that assigns recursively the index of the intervals to a real number $x \in [0,1]$. It should be noted that $S^{(i)}$ is uniquely determined by $\mathscr{L}$ and k. $S^{(0)}$ is mathematically interchangeable to $\mathscr{L}$.

(2) For each interval l_i, a MFNG $\mathscr{G}_k$ induces k-th order recursive partition such that $[0,1]$ is eventually partitioned by m^k intervals sampled from a generalized Bernoulli distribution,

$$P\{S^{(k)}(x) = i_1, i_2, ..., i_k\} = \Pi_{t=1}^{k} l_{i_t}, x \in [0,1] \tag{10}$$

(3) Distribute N nodes to m^k intervals arbitrarily. Define the linking probability measure to be $\mathscr{P}$ such that two nodes in interval l_i and interval l_j, respectively, are joined by a link with a probability $p_{i,j} \in \mathscr{P}$. The linking probability $\mathscr{P}$ is obtained by the k-th order tensorial product of the generative measure $\mathscr{P}_0$,

$$\mathscr{P} = P^{(k)} = P^{(k-1)} \otimes \mathscr{P}_0 \tag{11}$$

$$P^{(0)} = \mathscr{P}_0 \tag{12}$$

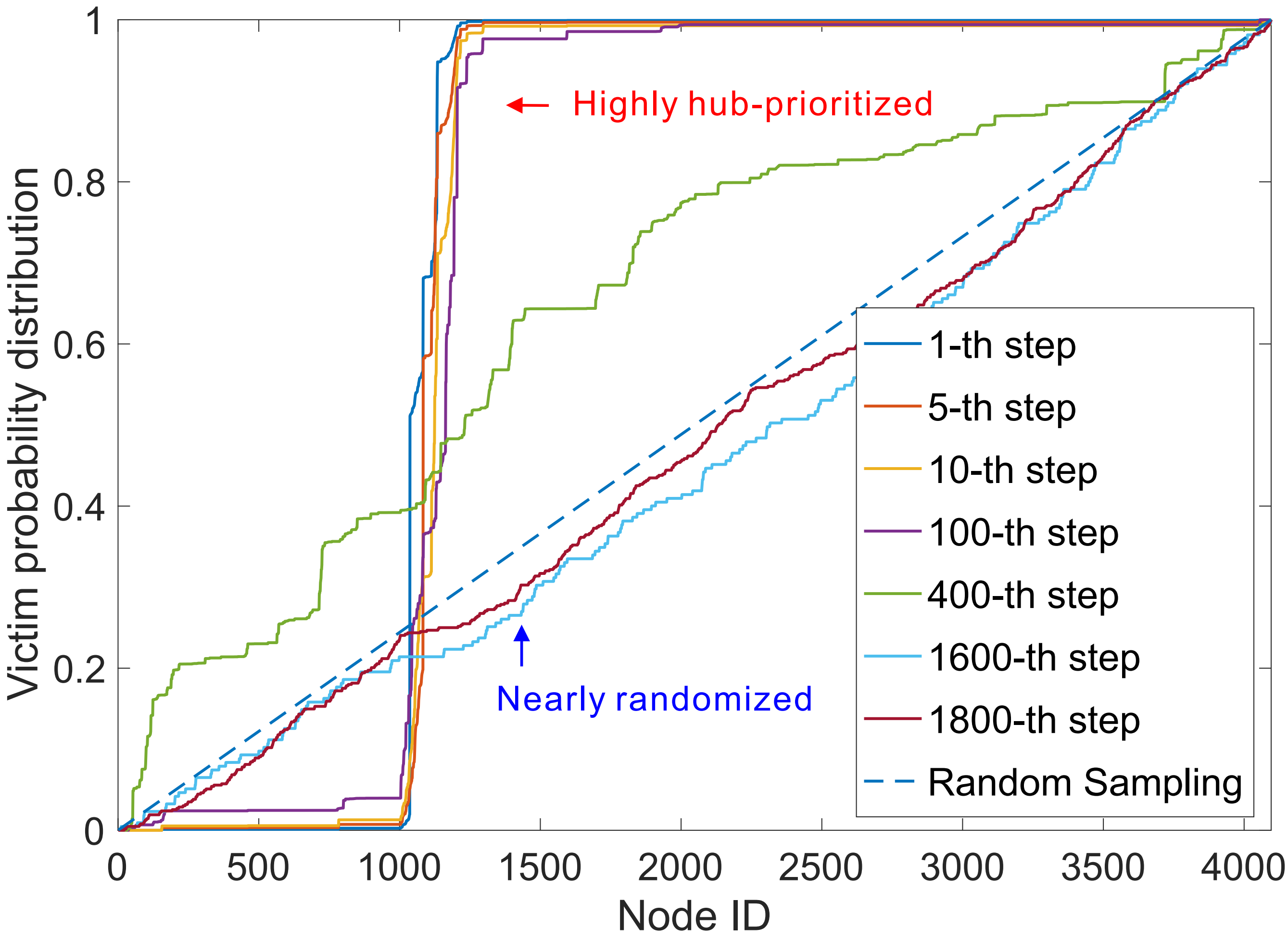

Supplementary Figure 6. Transitional behavior of interventions ($\alpha = 10$) on the human protein complex interaction network over time.

where $\otimes$ is the Kronecker product. A generative measure $\mathscr{P}_0$ is a m by m matrix with its entry $p_0(i,j) \leq 1$. Procedure (1-3) denotes a generative process to construct a network from a multi-fractal measure defined on the unit square. By construction, any pair of nodes lying at the same interval l_i links to a node in $_j$ with the same probability $p_{i,j} \in \mathscr{P}$. This construction is aligned with the network motif and clusters in real systems where nodes within same community share similar interacting patterns with nodes outside the community. Reversely, one can expect to generate such network motifs during the inference process by prioritizing the assignment of the same interval index to a particular group of missing nodes (e.g., by optimizing over mapping function ψ). This is critically important if such network motifs exist in the original network.

Throughout our experiments, we assume the partition $\mathscr{L}^{(k)}$ on the interval $[0,1]$ is equally sized and each corresponds to at most one node in the network. As a result, an entry $p_{i,j}(k)$ in $P^{(k)}$ can be simply understood as the linking probability between a pair of nodes i and j. We set m to be 2 with a proper choice of k. We keep these experimental setups for all our experiments.

Supplementary Note 5: Derivation of the gradient of log-likelihood function

The complete likelihood function given the network model $\mathscr{G}$ is defined by,

$$P(G_t, M_t, \psi, \pi | \mathscr{G}, \mathscr{A}) = (\Pi_{(i,j)\in E_0} p_{\psi(i),\psi(j)} + \Pi_{(i',j')\notin E_0}(1 - p_{\psi(i'),\psi(j')})) * \gamma \Pi_{s=0}^{t-1} A_\alpha(d(\pi^{-1}(s)), s) \tag{13}$$

The log-likelihood function LL is thus,

$$LL = \sum_{(i,j)\in E_0} \log(p_{\psi(i),\psi(j)}) + \sum_{(i',j')\notin E_0} \log(1 - p_{\psi(i'),\psi(j')}) + \sum_{s=0}^{t-1} \gamma A_\alpha(d(\pi^{-1}(s)), s) \tag{14}$$

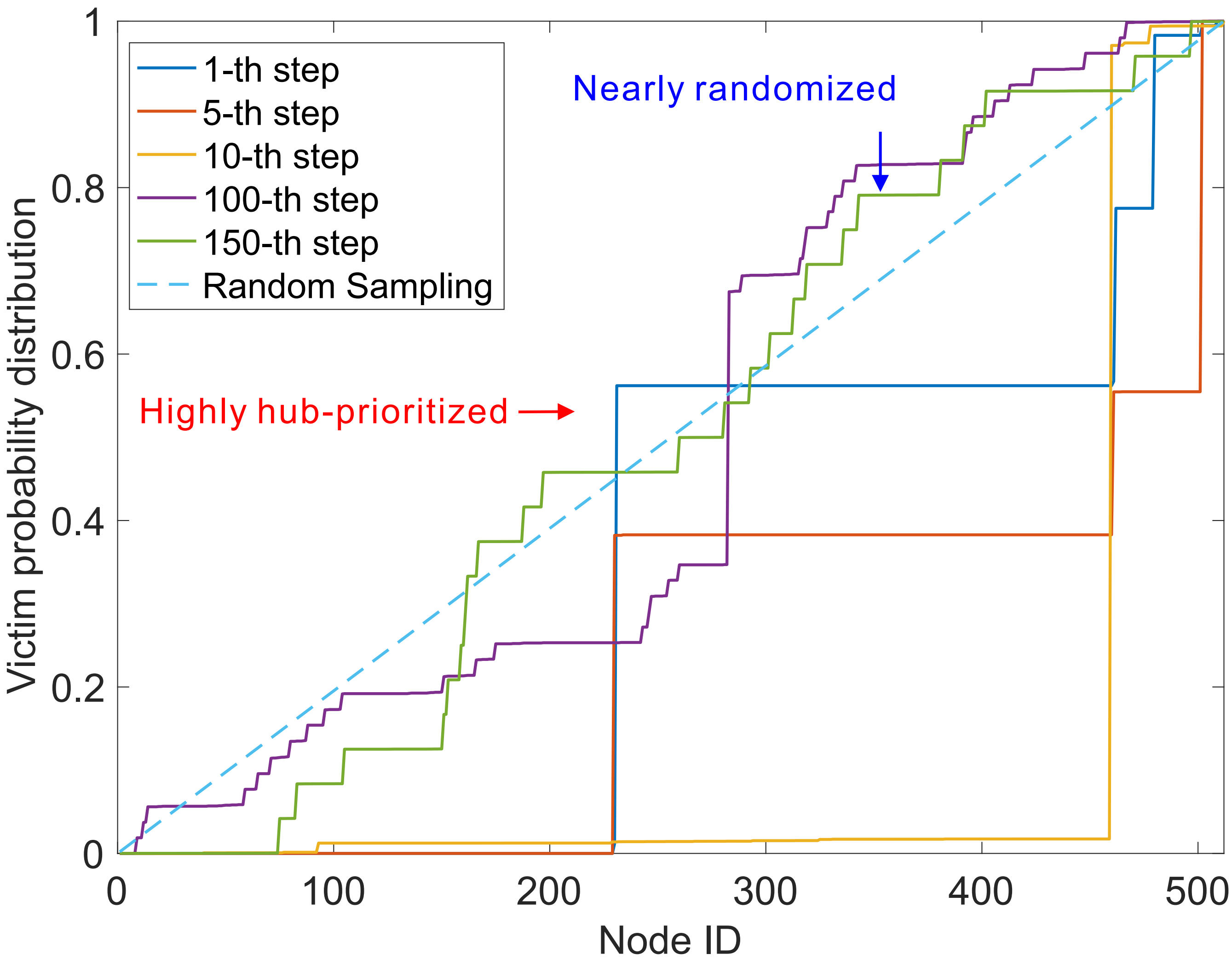

Supplementary Figure 7. Transitional behavior of interventions ($\alpha = 10$) on the human consensus brain connectome over time.

We notice the gradient of LL in terms of the generative measure p_0 does not depend on $\gamma A_\alpha(d(\pi^{-1}(s)), s)$. Therefore, we define LL' as,

$$\begin{aligned} LL' =& \sum_{(i,j)\in E_0} \log(p_{\psi(i),\psi(j)}) + \sum_{(i',j')\notin E_0} \log(1 - p_{\psi(i'),\psi(j')}) \\ =& \sum_{i,j} \log(1 - p_{\psi(i),\psi(j)}) + \sum_{(i,j)\in E_0} \log(\frac{p_{\psi(i),\psi(j)}}{1 - p_{\psi(i'),\psi(j')}}) \\ =& A_1 + A_2 \end{aligned} \tag{15}$$

Taking the derivative of the first term A, we have,

$$\frac{\partial A_1}{\partial p_0(u,v)} = -\sum_{i,j} t \frac{p_{\psi(i),\psi(j)}}{1 - p_{\psi(i),\psi(j)}} \frac{1}{p_0(u,v)} \tag{16}$$

where t is the number of $p_0(u,v)$ that $p_{\psi(i),\psi(j)}$ consists of (see Equation (22, 23) in Methods). Similarly, we have,

$$\frac{\partial A_2}{\partial p_0(u,v)} = \sum_{(i,j)\in E_0} \frac{1}{1 - p_{\psi(i),\psi(j)}} \frac{t}{p_0(u,v)} \tag{17}$$

```
The main entry of the code is EM_infer_attack_core_fb_opt_sampling.m

#---------------Input setup------------------------------
To run the reconstructor, need to prepare first the input

1.graph.mat which is a matlab graph object.

or

1.Adjacency matrix of the graph.

#--------------Configure your run------------------------
User panel is used to configure your run
#Decide the sample size
Burnin = 0;
N_samples = 10000;
N_samples_psi = 10000;
N_batches = 100;
N_opt_steps = 100;

#Parameters to tune the gradient descent (you can choose to use momentum by setting beta a non-zero value)
#lambda is the learning rate.
lambda = 10e7;
epsilon = 10e-6;

#How to initialize your graph guess
ini_method = 'random';
forced_degree_lb = 25;

#Parameters of attack model
alpha = 100;
beta = 0;
loss_percentage = 0.01;

#Parameter of the MFNG parameter
#The graph size := m_opt^(k_opt)
```

Supplementary Figure 8. Instruction to run the code.

Thus,

$$\frac{\partial LL}{\partial p_0(u,v)} = \frac{\partial A}{\partial p_0(u,v)} + \frac{\partial B}{\partial p_0(u,v)} \tag{18}$$

We have an important note here. Given a large number of samples, the amortized computational complexity of Equation (18) is $O(|E_0|)$. We notice Equation (16) does not depend on the original network G_0 as it is a function of upper/lower triangle of $\mathscr{P}$ for undirected networks (and $\mathscr{P}$ is symmetric) and the whole $\mathscr{P}$ for directed networks, irrespective of ψ. As a result, the gradient is a constant given the fixed generative measure p_0 and thus its computational can be amortized by all samples of $\{Z_t, \psi\}$. Equation (17) is clearly a function of $\{Z_t, \psi\}$ and it takes $O(|E_0|)$ to compute. Consequently, the amortized computation cost to calculate the gradient is $O(|E_0|)$.

Supplementary Note 6: Code tutorial

We have included the code tutorial as Supplementary Figure 8.

Supplementary References

1. Watts, D. J. & Strogatz, S. H. Collective dynamics of small-world networks. *nature* **393**, 440 (1998).

2. Bollobás, B. Random graphs. In *Modern graph theory*, 215–252 (Springer, 1998).

3. Humphries, M. D. & Gurney, K. Network small-world-ness: a quantitative method for determining canonical network equivalence. *PloS one* **3**, e0002051 (2008).

4. Newman, M. E., Strogatz, S. H. & Watts, D. J. Random graphs with arbitrary degree distributions and their applications. *Physical review E* **64**, 026118 (2001).

5. Cohen, R. & Havlin, S. Scale-free networks are ultrasmall. *Physical review letters* **90**, 058701 (2003).

6. Palla, G., Lovász, L. & Vicsek, T. Multifractal network generator. *Proceedings of the National Academy of Sciences* **107**, 7640–7645 (2010).
